# P-1086. In-vitro Susceptibility of Eravacycline against Enterococci including Daptomycin Non-susceptible and Linezolid Resistant Isolates

**DOI:** 10.1093/ofid/ofaf695.1281

**Published:** 2026-01-11

**Authors:** Hongshik Park, Nicholas Feola, Utsav Pandey, Guiqing wang, Abhay Dhand

**Affiliations:** New York Medical College, Valhalla, NY; Westchester Medical Center, Valhalla, New York; Kaiser Permanente, Los Angeles, California; NYU Langone Health, New York, New York; Westchester Medical Center, Valhalla, New York

## Abstract

**Results:**

A total of 52 nonduplicate DNSE *E. faecium* clinical isolates were analyzed in this study. Of these 2/52 were both LRE and DNSE (MIC range: 6 to > 256 μg/ml) and 48/52 were VRE. The MIC50 and MIC90 of eravacycline against combined resistant enterococcal isolates was 0.032 μg/ml and 0.25 μg/ml (range: 0.008 – 1μg/ml) respectively. For 2 DNSE and LRE isolates, eravacycline MICs ranged from 0.032 to 0.064 μg/ml.

**Conclusion:**

Eravacycline offers a therapeutic option for treatment of select infections caused by multi-drug resistant Vancomycin resistant, Daptomycin non-susceptible and Linezolid resistant enterococci. Based on its in-vitro susceptibility, eravacycline can also be considered as a daptomycin-sparing and/or carbapenem-sparing option for the treatment of polymicrobial intra-abdominal or wound infections caused by various resistant gram-positive and gram-negative organisms.

**Disclosures:**

Abhay Dhand, MD, Eurofins Viracor: Advisor/Consultant|Eurofins Viracor: Honoraria|Merck: Advisor/Consultant|Merck: Honoraria

**Background:**

Eravacyline is a fully synthetic next generation tetracycline with broad spectrum activity against multi-drug resistant gram-positive and gram-negative organisms. To address a rise in daptomycin non-susceptible enterococcus (DNSE) in our institution, we started an organism and syndrome-based treatment algorithm, where eravacycline was used to treat selected non-bacteremic, non-urinary tract polymicrobial or mono-microbial infections associated with vancomycin resistant enterococci (VRE). The aim of this study was to concurrently evaluate in- vitro antimicrobial susceptibility of eravacycline against enterococci with focus on daptomycin non-susceptible and/or linezolid resistant (LRE) clinical isolates.

**Methods:**

DNSE and LRE clinical isolates were recovered from patients in a tertiary medical center in suburban New York City from May 2013 through December 2019. Daptomycin susceptibility was determined by MicroScan WalkAway™ System and confirmed by E-test. Susceptibility of linezolid was performed by broth microdilution using the Sensititre™ panel. Eravacycline minimum inhibitory concentration (MIC)s were obtained by E-test in accordance with package insert of the manufacturer.

